# On Our True and False 'Knowledge' of the Physiological Character and Action of the Red-Blood Corpuscles

**Published:** 1868-01

**Authors:** Rufus King Browne


(To be Continued.)

---

### ARTICLE II.

### *On our True and False 'Knowledge' of the Physiological Character and Action of the Red-Blood Corpuscles.*

By Rufus King Browne, M.D.

It is still said to be doubtful whether the corpuscles consist of red-liquid enclosed by a membrane or not, but recent researches have left not the slighest basis for a reasonable doubt upon that point, but unequivocally and imperatively ratify every observation of its non-membranous form, by showing precisely what function it performs, and thus making it indubitably evident, that the existence of membrane or wall is entirely incompatible with the performance of that function. This function has long been suspected to be the carrying of oxygen throughout the animal body. During its passage through the lungs, the whole blood, as every one knows, taught by innumerable experiments which establish the fact, loses carbonic acid and takes up oxygen.

Every one hundred volumes of blood, which enter the lungs are capable, according to Claude Bernard, of absorbing twenty-one volumes of oxygen. This is about seven times as much as an equal quantity of water could dissolve, and it has been shown that serum which differs but slightly from the blood fluid, was hardly superior to water in this respect. Consequently, it is evident that the great mass of the oxygen must be attracted by the blood corpuscle, and that it surrenders nothing of itself with the oxygen it delivers.

It has therefore been generally assumed, though it was not proved, that the substance of the corpuscle was capable of combining with oxygen in the lungs, and afterward of giving out that oxygen again, in small doses, to the substances it came in contact with. This has now been unequivocally demonstrated to be the case, and it is exclusively upon this demonstration we rely, to establish 1st, that the red blood-corpuscles alone appropriate the oxygen. 2nd, that they yield this to the plasma in the round of the circulation, and 3d, that they return to the lungs to take a fresh supply, which again is distributed and so on : and, lastly, that the characteristic action of the corpuscles in this transaction is one which demonstrates its vivid character and function.

Hoppe-Seyler first recorded the curious fact, that when a ray of white light passes through a solution of red-blood, and is afterwards decomposed by a prism, two dark bands make their appearance in the green portion of the spectrum. Stokes repeated and verified this observation, and made it the starting point of a new, exhaustive and decisive train of research.

He treated a solution of blood *corpuscles* with an alkaline reducing agent, and observed that its color instantly changed, from a scarlet to purple red, the hue of venous blood. On examining the spectrum of this, he found that the two dark lines had disappeared, and that a single line only, intermediate in position between them, had became visible in the spectrum. On shaking the tube with air, the scarlet color, and the two lines at once returned, but, after a few minutes again *disappeared*, and this could be repeated many times. Hence it was evident that the scarlet arterial blood repeatedly lost its oxygen to the reducing agent, and subsequently recovered it again, then shaken from the air ; thus undergoing the precise variation in color, the change from light to dark, through which it regularly passes in the circulation. This experiment shows that the purple of venous blood is *not* due to the existence in the red-globule of carbonic acid gas,—neither from the oxidation of its own substance, as embodied in one of our suppositions, nor from its taking up carbonic acid gas *not* of its own substance, whether from within or from without the blood. It shows, further that there are not *two* changes of color accurately stated, but only one, namely, that from purple to scarlet, and that is dependent on the gain of oxygen. It shows also that it is the *loss* of the oxygen it absorbed, and not the *absorption* of carbonic acid, which causes the cessation of the scarlet.*

This matter of the red blood corpuscle, always called its coloring matter,—is different from the so-called hematin, which is obtained by artificial means, from the blood. In the oxidized—the SCARLET state—it is distinguished as *scarlet cruorine*, and in its second state, it is *purple cruorine*. The two conditions are simply those in which it holds different quantities of oxygen. It it hardly necessary to point out how very striking and conclusive an explanation these facts afford of the oxygen-carrying capacity of the red blood corpuscles. They remove all obscurity as to precisely what constituent of the blood it is which performs this office, and they demonstrate that it is exclusively those bodies which take up the oxygen. In the

---

* This experiment is very instructive, for it shows the *hue* of venous blood, or its corpuscles, is not a new *color*, but is simply the organic hue of its substance and form, which is *not* another color, but the basis of the change to scarlet caused by the impletion of oxygen.

lungs the substance of the blood corpuscle, the *purple cruorine* of venous blood—takes up oxygen and becomes scarlet or arterial cruorine, and in the systemic circulation this oxygen is given up to the plasma, and the substance of the globule the *cruorine*, passes into the purple state.

But of this latter fact, a second demonstration actually exists in another experiment of Stokes. And this experiment shows that the blood globules have also the power of discharging their oxygen into the midst of the other blood substances, i. e. the plasma. In this experiment a solution of the blood corpuscles from arterial blood—a solution that is of scarlet cruorine—when excluded from the air and the access of oxygen, shortly *reduced itself*, or parted with its oxygen to the serum and became purple cruorine. But some reader may be inclined to inquire, do these experiments really show that it is the corpuscles and not the plasma, which take up the oxygen? To this we answer that proofs, not alone perfectly conclusive, had led us to the belief, that the former took up the oxygen at the lungs, long before these actual demonstrations of the fact.

First, in these demonstrations it is shown that the substance of the corpuscle *will* and invariably does take up the oxygen from the air quickly, almost instantly, and gives it out *some time* afterward. This fact perfectly accords with the physiological phenomena of their taking up oxygen in the body, from the air in the lungs. Here the red blood corpuscles literally crowd the pulmonary capillaries, almost to their distension and the exclusion of the plasma, and when coming directly from them, are found to be abundantly supplied with oxygen.

Second, this oxygen if looked for in the same corpuscles of venous blood is wanting ; and this alternation may be repeated any number of times. This fact was re-demonstrated by opening the tube and shaking it with air ; when the scarlet color returned, and with it the two lines of the spectrum. In these experiments it is proven that the scarlet cruorine is capable of yielding the oxygen it holds to a portion either of the serum or the plasma, either of them hardly superior to water in the amount of oxygen *they* can *take up*. This action of the red-corpuscle,—of its substance the cruorine, in taking up under certain conditions, the oxygen and giving it up afterwards, to the substances of the liquor sanguinis, is one of a character—(give and take)—so invariably characteristic of organized *animal substances and processes*, that I must particularly dwell upon it.

We have seen that the notable phenomena of change of color in the blood, makes or signalizes a change in the substance of the globule, due to its appropriating the oxygen of the air, but that this change *does not last long*; that its duration is only up to a certain point in the blood channels and that from thence until the globules resume their situation, in the lung capillaries, the change was remitted. Now, here is a plain statement of a *fact*, and looking at it in the ordinary way, that is, without any exercise of rational intelligence, as a horse regards the landscape, or the sky, or his fellow horse without the slightest intelligence of the truth in either case not *seen* by the eye, we would be ready to say, that there was no difficulty in understanding and explaining the phenomena, in accordance with the ordinary theories of oxidation, and that the oxygen had oxidized the globule, and afterward left it. But then this statement plainly proves how total our *un*-intelligence is about the phenomenon,—how completely we leave unappreciated and mentally unseen, the true character of the transaction. The statement we have supposed to be made, is in two halves neither one of which can possibly be true if the other is. The one supposes the access of the oxygen to be oxidation. But the second part of the supposition is that the oxygen disengages itself. Now the impossibility of this second occurrence, consistently with the first as oxidation, proves the fallacy equally of both parts of the supposition. Oxidation presupposes the *union* of oxygen with particles of some other substance.

This, and nothing else, is what determines the exact character of oxidation. Now what kind of oxidation is it in which this union does *not* take place? What kind of oxidation is it in which the very characteristic of the phenomenon lies in the complete after separation or disengagement of the oxygen, subsequent to its engagement in the lungs? Can it be rationally said that this disengagement is an *act of* the oxygen? Can it indeed be said, that the act of taking it up and discharging it, has anything in it characteristic of oxidation except the physical character of the oxygen? Of course in saying that the case in question is not one of oxidation, we do not mean a negative, or, that it is a case in which no phenomenon, in which oxygen takes a part occurs, for instance, a case where oxygen is present with other substances subject to chemical change, and yet takes no part in changing them. No, what we mean, plainly enough is that admitting oxygen to *take part* in the process of arterialization, it yet performs no such part as in *oxidation*.

Undoubtedly, arterialization implies, *not* a change in the substance of the oxygen,—and that it takes part in the transaction, as it takes part in oxidation; but what it shows in which it differs from oxidation is this, that its power of appropriating to itself, or uniting with, at the expense of the integrity of their substance, other inorganic substances, is not in force on the substance of the globule, because unlike others, that substance has itself an *organic* power; and this latter power it is which provides that the peculiar activity of the oxygen, which ordinarily ultimates in oxidation shall be neutralized. But the difference we here point out is not that between two different physical traits or characters, but a difference between an *organic character on the one side, and of a physical* character on the other. But the reader may enquire, whether the oxygen is really disengaged from the globules. The experiments already cited prove that as venous cruorine or corpuscle substance will take up again, and again many times, oxygen from the air in contact with it, becoming each time arterial cruorine, so the latter if completely excluded from the air will soon give up its oxygen to the serum, thereby becoming venous cruorine. But this disengagement now questioned, has been all along explicitly affirmed by the supposition that the globules yield oxygen to the tissues. That they *yield it*, is as explicitly stated in this long-credited supposition, as it is by us. But we have been forced to abandon the idea, *that it is* to the *tissues* the oxygen is yielded. For the nature of the case is such that the tissues cannot receive it from the globule as transferred by contact, but only *from* the plasma or serum,—the exudate which *does* permeate the tissue. Our statement is the same as the supposition long existing that the globules do yield their oxygen, on their round, but rather than that they do so *to the tissues*, we say they yield it regularly throughout their round to the plasma,—the fluid in which they are suspended. The running anatomical and physiological conditions are such, as to show our statement to be the truth, for it is impossible that the globules can leave in their track in their rapid motion, a train of particles of oxygen, free to penetrate the sides of the vessels and permeate the tissues to the right and left, uncombined.

Such a notion belongs to a day of study, when our intelligence had not yet *come to the birth on* the subject. It implies what we know is not true, namely, that the oxygen leaves the corpuscles, traverses the walls of the blood-vessels, and arrives at the comparatively distant fibres of tissue, in an *un*combined state. Whereas to the least exercise of intelligence it is plain that the substances of the liquor sanguinis are in perfect contact with the globules, the former actually forming a coating for each of the latter, and the disengagement of the oxygen must be to the substances in the plasma. However, it has been demonstrated, that there is no *free* oxygen in the vessels, since pyrogallic acid, which is notable for combining freely with free oxygen, when it is injected into the veins, will pass out of them without undergoing oxidation. This actual delivery by the corpuscle of oxygen, elsewhere than where they received it, is implied in every supposition.

But to return to the heart of our subject. This transaction of the globules, is one in which they are the real *agents*, in which they do the *work*—a work which is similar in its organic character to the appropriating and discharging work of the various anatomical elements of the organism, and most notably of its cellular elements. This consists in their *doing* something and not in their being acted upon by some disintegrating substance, whose character lies in its *physical power*. The distinction between these two reaches its highest, in the blood corpuscles. Accordingly there is nothing at all beyond our present knowledge, to an active intelligence, in the assertion, that while the oxygen contributes its physical substance, and the ingoing and outcoming are its physical conditions, the globules do the specific organic work of arterialization. They appropriate the oxygen and yield it with equal facility. The liquor sanguinis cannot do this but is dependent on the globules for all it has. And the physiological relation of the two is such as to show that this could not possibly be done in any other way. Estimating the mere fraction of oxygen the plasma by itself in quantity will take up, as compared with the corpuscles, and that in the capillaries of the lungs the proportion of plasma to globules is much less than elsewhere, no supposition could be more distant from the truth, than that which supposes "the plasma to first take up the oxygen and then give it to the corpuscles."

[*Flint. Hum. Phy.*—"The plasma *first* takes up the oxygen and gives it to the globules."]

What can all this be, but proof actual and positive, that it is the globules themselves which take up this oxygen, and give it out again to the fluid they are suspended in, somewhere in the round of that fluid between the points in the circuit, where they are found to have it, namely, the arteries, and where again they are found to have lost or *discharged* it to the fluid in which they are suspended, namely, the plasma, to combine with oxidable materials, therein. What other reasonable notion do the facts admit of? None whatever! Is it for instance conceivable that the fluid of the blood takes it up in the lungs, immediately gives it to the globules to be again instantly surrendered by them to that fluid.

The bare supposition is absurd and contradictory; to say nothing of the facts being precisely otherwise. Shall we maintain the supposition, both prohibited by facts, and contradicted by *other* suppositions, that the red-blood globule does not *disengage its oxygen*, but retains it to the last, becoming in part oxidized, or decomposed with loss of substance,—or carbonic acid-*ized*? In that case let us confront *this* with another supposition, viz.: that the red-blood globule does lose or deliver oxygen in its capillary round and "*takes up* carbonic acid;" or that other supposition that the red blood corpuscle "consumes oxygen and "*gives off*" carbonic acid:" or, what explicitly contradicts this, namely, the other supposition, that the venous color of the red-globule is due to the carbonic acid of the blood, which seeing that the color is due to the corpuscle must according to this supposition be in them,—the latter according to the last prior supposition being what it "gives off" in the blood. All these we discover have the merit of being in flagrant contradiction each of every other. Still more the supposition relating to the corpuscle giving off carbonic acid, can *not* mean given off in the lungs, for another supposition states that the carbonic acid *there* is displaced from the plasma by the oxygen, which plasma is said to "first take up the oxygen and then give it to the globules," which latter according to this supposition, arive at the lungs without having carbonic acid.

The *fact* being, that the plasma cannot take up more than 1-15 of a supply of oxygen for the globules. What do we make of the fact that they take oxygen up instantly and subsequently discharge it much *less quickly*, as shown in the experiments? I emphasized the word discharged above, for what in my estimation demands the most lively and absorbing appreciation is, the part directly played by the globule, as shown by these experiments, in this transaction. For this is a part in which the globule is not the *patient*, but the true agent.

The act is invariably described as one of "oxidation," and it is owing to an inveterate, because wholly inconsiderate habit, of pronouncing it one of "oxidation," which designates it as a *chemical* process,—that we have such very infirm notions about it. This may have been excusable while we were wholly misinformed as to precisely in what connection the oxygen entered the system, and by what substances it was transported to the tissue supplying blood; while indeed, we thought the oxygen upon reaching the blood, by a process of "diffusion," entered at once upon a course of *chemical action*.

(To be continued.)

---

